# Supplementary material for: Comparative Analysis and Structural Modeling of Elaeis oleifera FAD2, a Fatty Acid Desaturase Involved in Unsaturated Fatty Acid Composition of American Oil Palm
Source: Biology (Basel). 2022 Mar 30;11(4):529. doi: 10.3390/biology11040529 (PMC9032008; doi:10.3390/biology11040529)
Supplement: Supplementary file 1 [file biology-11-00529-s001.zip › biology-1588326-supplementary.pdf]

Supplementary Materials

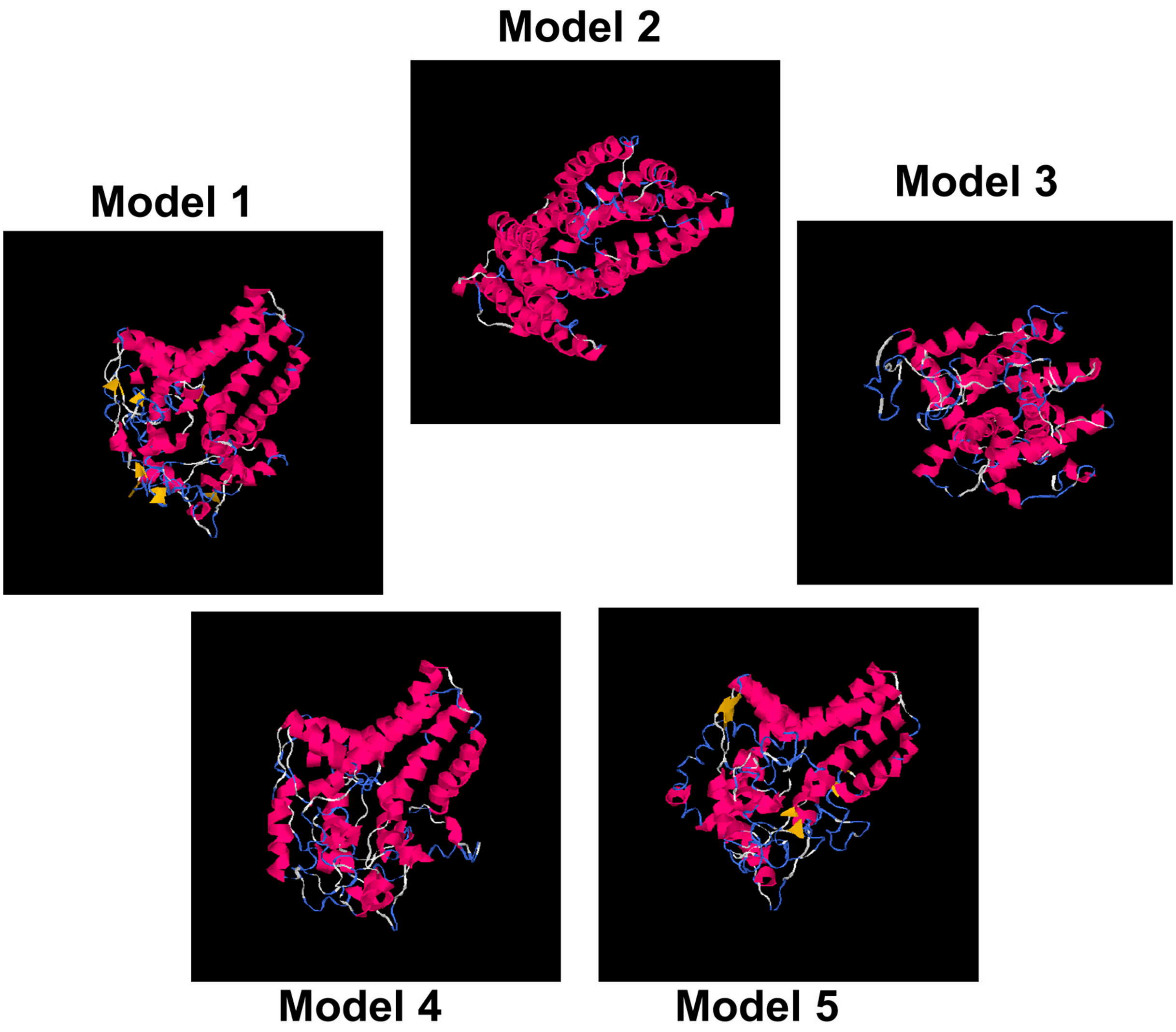

Figure S1. Five models were predicted using the I-TASSER tool.

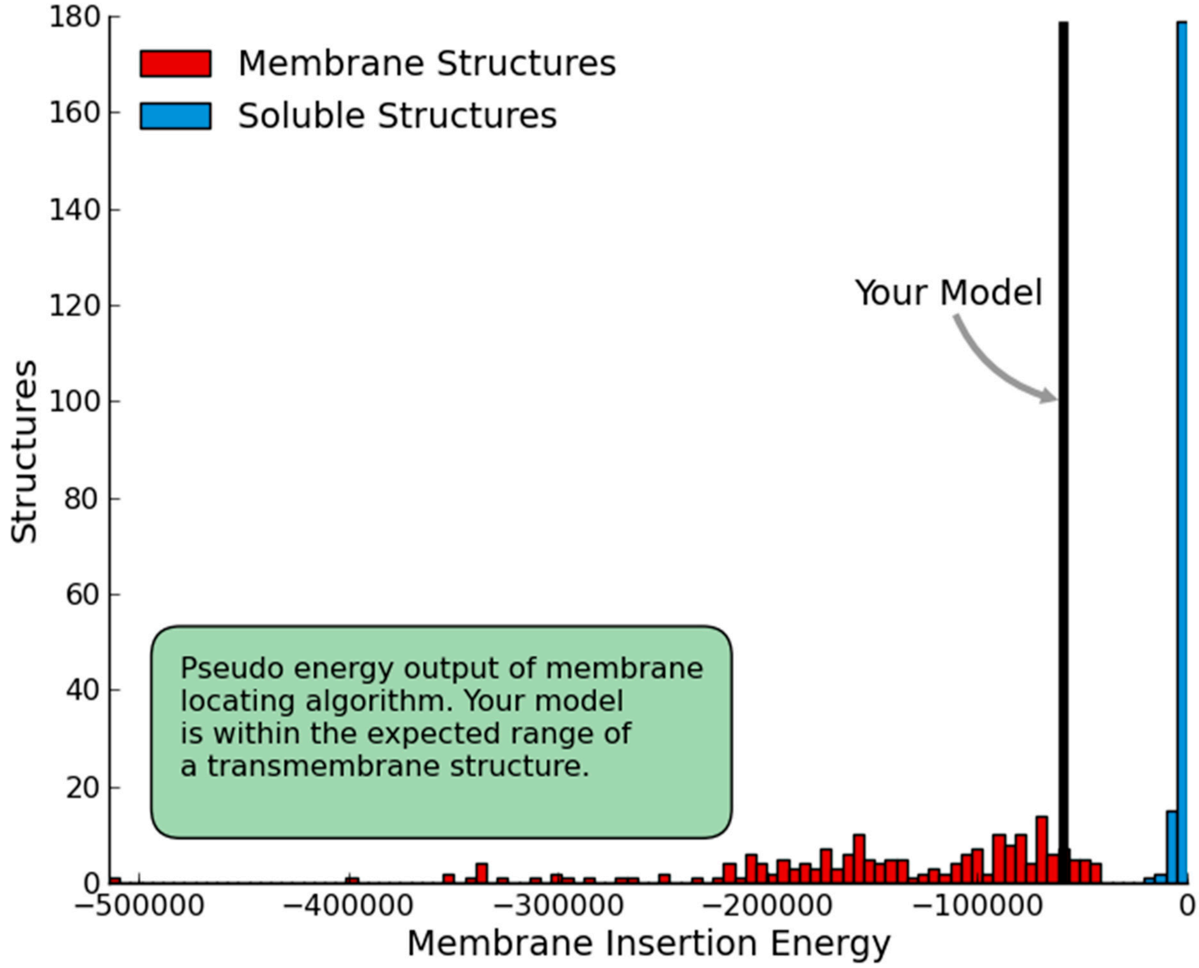

Figure S2. Energy minimization using OPLS-AA was performed on the initial structure obtained from I-TASSER to correct any bond-clashes and refine the stereochemistry of the predicted best protein model.
